# Evaluation of Multiplex loop-mediated isothermal amplification assay for the detection of *Mycobacterium tuberculosis* complex from clinically suspected cases of pulmonary tuberculosis

**DOI:** 10.1016/j.heliyon.2024.e39847

**Published:** 2024-10-24

**Authors:** Md Zaber, Fahmida Hoque, Ishraque Monir Paean, Shirin Tarafder

**Affiliations:** a Department of Microbiology, Gazi Medical College, Khulna, Bangladesh; b Department of Microbiology, Sheikh Hasina Medical College, Jamalpur, Bangladesh; c Armed Forces Medical College, Bangladesh; d Bangabandhu Sheikh Mujib Medical University (BSMMU), Dhaka, 1000, Bangladesh

**Keywords:** TB-LAMP, Pulmonary tuberculosis, MTBC, MTB/NTM qPCR, Non-tuberculous mycobacteria

## Abstract

Tuberculosis (TB) is the second leading cause of death from a single infectious agent worldwide. Bangladesh ranks 7th among the 30 high TB burdened countries in the world. Accurate detection of *Mycobacterium tuberculosis* complex (MTBC) is challenging for developing countries as most of the resource poor settings are not suitable to perform molecular techniques.

The purpose of the study was to compare the multiplex TB-LAMP assay with MTB/NTM qPCR, culture, Z-N staining, and fluorescence microscopy in order to assess the effectiveness of the LAMP assay for detecting cases of pulmonary tuberculosis.

This research work was done from March 2022 to February 2023. Fulfilling the inclusion criteria 130 sputum samples were collected. TB-LAMP assay, qPCR, culture in L-J media, Z-N staining and fluorescence microscopy were performed.

Out of 130 samples qPCR detected MTBC in 56.92 % cases and TB-LAMP detected 53.85 %. MTBC was detected by culture 46.15 %, by Fluorescence microscopy 40.77 % and Z-N staining 36.92 %. TB-LAMP detected 16.93 % more cases than Z-N staining and 13.08 % more cases than fluorescence microscopy. The sensitivity, specificity, positive, and negative predictive values of multiplex-LAMP assay were 95 %, 81.4 %, 81.4 % and 95 % respectively considering culture as a gold-standard. MTBC negative culture samples (18.57 %) showed positivity by LAMP assay as well as by qPCR. This study detected 7.69 % non-tuberculous mycobacteria (NTM) by qPCR. All NTM positive samples were negative by TB-LAMP.

TB-LAMP is an easy to perform, cost-effective, reliable assay with high sensitivity and specificity. World Health Organization recommended TB-LAMP as a rapid molecular test for rapid detection of tuberculosis and as replacement of microscopy in resource poor settings/hard to reach areas. Bangladesh being a high TB burden country it is essential to implement TB-LAMP to achieve End TB Strategy by 2035.

## Highlights

• Multiplex TB-LAMP assay detects more cases than fluorescence microscopy.
• Overall performance of multiplex TB-LAMP assay is almost similar to that of RT-PCR.
• Multiplex TB-LAMP assay is positive in all smear positive and culture positive case.
• TB-LAMP assay can be a replacement of smear microscopy to detect MTBC.

## 1 Introduction

Tuberculosis (TB) is a communicable disease caused by *Mycobacterium tuberculosis* (MTB) remains a major public health problem worldwide despite the availability of effective treatment for decades.

Worldwide approximately 10.6 million people became affected with TB and 1.6 million people died of tuberculosis in 2021 [1]. Bangladesh is among the top 8 countries accounting for two-thirds of the global TB burden. Tuberculosis is a massive threat for control of TB nationally in Bangladesh with an incidence rate of 221 per 1,00,000 and 38,000 annual deaths [2]. Most cases cannot be diagnosed due to the unavailability of reliable diagnostic tests at peripheral settings or the poor sensitivity and specificity of available tests. Therefore, to achieve the End TB Strategy of WHO by 2035, early detection of MTBC is important to initiate the treatment as well as to control the transmission of tuberculosis in the community [3].

However, current microbiological tests for detecting pulmonary TB have their known limitations, especially in peripheral level/hard-to-reach areas. Microscopy of sputum is rapid and of low cost but has no sensitivity and specificity. It cannot differentiate MTB and nontuberculous mycobacteria (NTM). Sputum smear microscopy shows sensitivity which is from 50 to 60 % in pulmonary TB [4]. Fluorescence microscopy has improved sensitivity by 10 % compared with Ziehl-Neelsen staining (Z-N staining) which can be performed rapidly and is available now in most of the centers of TB control programs [5]. The gold standard for diagnosis of TB is culture but it takes more time and is susceptible to contamination. In L-J media growth occurs in 4–6 weeks and in automated system 1–6 weeks [6]. Molecular detection methods offer a rapid detection of pathogens [7]. However, most of these methods are costly and require sophisticated equipment. Real-time PCR requires a thermocycler, equipment for result analysis and expert manpower. Recently developed Nucleic Acid Amplification Test (NAAT), Gene Xpert MTB/RIF can detect MTBC with rifampicin-resistance in sputum samples without processing. However initial investments in the equipment and subsequent maintenance remain quite expensive [8]. It also requires a continuous power supply which is quite challenging to achieve in resource-poor settings/hard-to-reach areas of developing countries like Bangladesh.

WHO endorsed a molecular method, TB-LAMP assay for diagnosis of pulmonary TB as an alternative to smear microscopy. There are several advantages of this method: it does not require complicated equipment, unprocessed samples can be used for DNA amplification, amplified product is visible by naked eye, risk of contamination is less [9]. The sensitivities of TB-LAMP assay have been evaluated by Several studies [[10], [11], [12], [13]]. Earlier research employed in-house kits. A few research compared the TB-LAMP assay to both LED microscopy and AFB staining. There is no available published data yet, regarding the evaluation of Multiplex TB-LAMP assay for detection of MTBC in Bangladesh. Bangladesh is a low middle income and high TB burden country. To increase case detection for eliminating TB the country needs to replace microscopy with molecular diagnostic method as the initial diagnostic test. As TB-LAMP is less costly and robust to harsh condition it can be used to replace the microscopy in peripheral laboratories. This evaluation of TB-LAMP assay on Bangladeshi people will help National TB control program (NTP) in Bangladesh to choose a molecular tool for replacement of microscopy.

Therefore, the study aimed to evaluate the multiplex loop-mediated isothermal amplification (LAMP) assay for detecting MTBC in sputum collected from presumptive pulmonary tuberculosis cases & compare it with MTB/NTM real-time polymerase chain reaction (qPCR), culture, Z-N staining and fluorescence microscopy, thereby to see its feasibility for implementing in resource-poor settings/hard to reach areas of Bangladesh.

## 2 Material and methods

### *2.1* Study design

This is a cross-sectional study. The study used the STROBE cross-sectional reporting guidelines [14].

### 2.2 Study settings

The study was carried out at the Department of Microbiology & Immunology, Bangabandhu Sheikh Mujib Medical University (BSMMU), Dhaka, Bangladesh and the National Tuberculosis Reference Laboratory, Mohakhali, Dhaka, Bangladesh from March 2022 to February 2023.

### 2.3 Sample collection

Sputum was collected according to the inclusion and exclusion criteria from 130 clinically *s*uspected Pulmonary tuberculosis patients attended at Directly observed treatment, short-course (DOTS) corner of BSMMU, National Institute of Disease of the Chest and Hospital (NIDCH), Mohakhali, Dhaka, 250 Bedded TB Hospital, Shyamoli, Dhaka. Microbiological investigations Z-N staining, Fluorescence microscopy, mycobacterial culture and TB-LAMP were performed. The outcome of these tests was observed and marked as MTBC positive, MTBC negative and NTM positive.

### 2.4 Inclusion criteria (National guideline and operational manual for Tuberculosis,2021)

According to the National Tuberculosis Control Programme (NTP) of Bangladesh, inclusion criteria for clinically suspected pulmonary TB patients are those who have one or more of the following symptoms in addition to Cough (especially if lasting for 2 weeks or longer, with or without production of sputum and despite the administration of a broad-spectrum antibiotic without antitubercular action [15].

• Chest pain
• Breathlessness
• Coughing up blood (hemoptysis)
• Fever
• Loss of appetite
• Unexplained weight loss
• Night sweats

The following exclusion criteria have been applied to the data from the participants.

1) Confirmed case of pulmonary tuberculosis
2) Taking anti-TB drugs

### 2.5 Data collection methods

Relevant data was obtained directly from the participants and results were recorded in a predesigned data record sheet. The collected data were checked for whether it is adequate, relevant, consistent or not.

### 2.6 Sample size calculation

$$smple\;size\;\left(n\right)\;based\;on\;specifitcity=\frac{Z_{1-\alpha /2}^{2}\times S_{p}\times \left(1-S_{p}\right)}{L^{2}\times \left(1-prevalence\right)}\text{,}$$

Where *n* = required sample size,

*S*_*P*_ = anticipated specificity, On the basis of previous article we assumed the specificity as = 90 % = 0 0.9 [16]

*α* = size of the critical region (1 – *α* is the confidence level),

*z*_1-α/2_ = standard normal deviate corresponding to the specified size of the critical region (α), = 1.96.

*L* = absolute precision desired on either side (half-width of the confidence interval) of sensitivity or specificity = 0.07.

This study considered the prevalence as 40.68 % [17].

n= (1.96)2 × .9 × 0.1÷(0.07)2 × (1-0.4068) = 121.06

So, minimum sample size was 122. This study had taken 130 samples considering the availability of the sample.

### 2.7 Microscopy and culture

AFB staining and Auramine O staining were done from the smear prepared from the sputum. The findings was reported according to WHO-recommended guidelines (WHO,1998). Decontamination of the sample were done by n-acetyl-L-cysteine-sodium hydroxide (NALC-NaOH) method then inoculated in L-J media as per standard literature [18]. The positive culture growth was confirmed for MTBC by SD bioline MPT64 Ag kit [19]. NTM growth was defined as positive culture growth that was determined to be negative by the SD Bioline MPT64 Ag kit [16].

### 2.8 Real-time PCR

Real-time PCR was done by GENMARK MTB/NTM Detection Kit For Real-Time PCR (www.genmark.com.tr) following instructions of the manufacturer. GENMARK MTB/NTM Real Time PCR Detection Kit is designed for the detection of MTBC and NTM by targeting IS6110 (MTBC) and 16S RNA (pan-Mycobacterium) genes. DNA extraction was done by Extraction Solution that was available in the MTB Real-Time Detection kit that can detect both MTBC and NTM.

### 2.9 TB-LAMP assay

TB-LAMP assay was done by Loopamp™ PURE DNA Extraction Kit and Loopamp MTBC Detection Kit (Eiken, Japan). Primers used here have been designed in the gyrase subunit B (gyrB) and Insertion sequence IS6110 (IS) region of the MTBC genome DNA. The sputum sample (60 μL) was taken into the heating tube and incubated at 95 °C for 5 min. The absorbent tube and heating tube was tightened, mixed the contents by inverting the tubes five times and then squeezed into LAMP reaction tubes and then incubated at 65 °C. Final results were interpreted by seeing the green fluorescence. All these procedures were performed by using the HumaLoopT instrument, provided by HUMAN Diagnostic Worldwide, Wiesbaden Germany. Fourteen sputum samples were tested at a time. To see the positivity of the test, positive control and negative controls were used.

### 2.10 Statistical analysis

All the parameters were calculated using IBM SPSS (version-26) for Microsoft Windows software. Chi-square test was performed to calculate P value. Significance was determined when P value was <0.05. Agreement between the two tests was measured by the kappa coefficient. Sensitivity, specificity, positive predictive value (PPV) and negative predictive value (NPV) of different methods for detection of MTBC were calculated taking culture as a reference standard.

## 3 Results

### *3.1* Positive results of TB-LAMP assay, qPCR for MTBC, Z-N staining, fluorescence microscopy and culture for MTBC

**Fig. 1:**
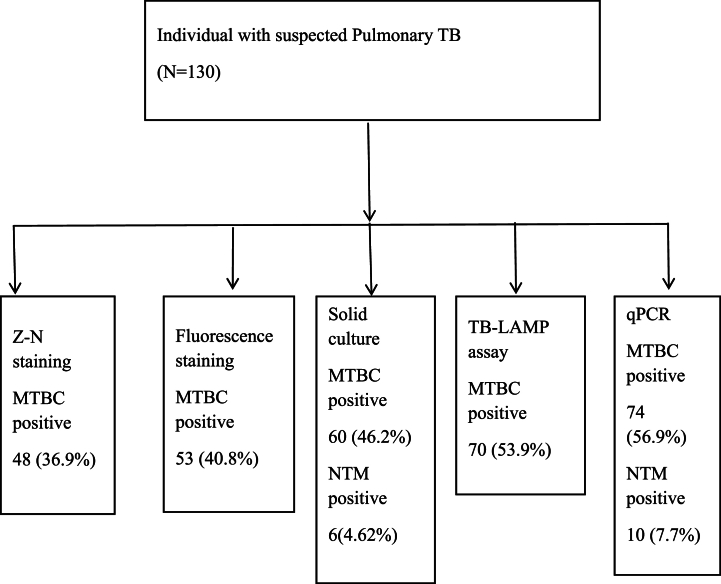
Flow chart of patient enrolment and diagnostic tests.

Fulfilling the inclusion criteria 130 sputum samples were collected. Among those 75(70 %) were from male and 39 (30 %) were from female, with age ranging from 18 to 78 years. Of the 130 sputum samples, TB-LAMP assay was positive in 70 (53.9 %) cases, cultures were Positive for MTBC in 60(56.2 %) cases, microscopy with Z-N staining was positive in 48(36.9 %) cases, Fluorescence microscopy with Auramine-O staining was positive in 53(40.8 %) and qPCR for MTBC was positive in 74 (56.9 %) cases (Fig-1). TB-LAMP assay detected 22 (16.9 %) cases more than Z-N staining 17 (13.1 %) cases more than fluorescence microscopy and 10 (7.7 %) cases more than culture.

### *3.2* Comparison of TB –LAMP assay with both Z-N staining grade and fluorescence microscopy grade

The positive rate of TB-LAMP assay increased with both Z-N staining and Fluorescence microscopy grade. TB-LAMP assay was positive in 10 (76.9 %), 23 (95.8 %), 9 (100 %), and 2 (100 %) of scanty, 1+, 2 +, and 3 + samples of Z-N staining respectively (Table-1). TB-LAMP assay was positive in 12 (80 %), 24 (96 %), 11 (100 %), and 2 (100 %) of scanty, 1+, 2+, and 3+ samples of fluorescence microscopy respectively (Table-1).

**Table 1:** Comparison of TB –LAMP assay with both microscopy grades.

Microscopy Grade	LAMP positive n (%)	LAMP negative n (%)	Total
**Z-N staining**			
Negative	26/82 (31.7 %)	56/82 (68.3 %)	82
Scanty	10/13 (76.9 %)	3/13 (23.1 %)	13
1+	23/24 (95.8 %)	1/24 (4.2 %)	24
2+	9/9 (100 %)	0/9 (0.0 %)	9
3+	2/2 (100 %)	0/2 (0.0 %)	2
**Fluorescence microscopy**			
Negative	21/77 (27.3 %)	56/77 (72.7 %)	77
Scanty	12/15(80.0 %)	3/15 (20.0 %)	15
1+	24/25 (96.0 %)	1/25 (4.0 %)	25
2+	11/11 (100 %)	0/11 (0.0 %)	11
3+	2/2 (100 %)	0/2 (0.0 %)	2

### *3.3* Comparison of results of TB-LAMP assay with Z-N staining and culture results

While comparing with both Z-N staining positive and Culture positive 38 cases, all cases were positive by LAMP assay (100 %). Twenty-two (22) cases were Z-N staining negative but Culture positive. Out of these 22 cases, 19 (86.4 %) cases were positive by LAMP assay. In both Z-N staining and Culture-negative 60 cases, 7 (11.7 %) cases were LAMP assay positive (Table-2).

**Table 2:** Comparison of results of TB-LAMP assay with microscopy and culture results.

Microscopy	Culture	Positivity rate of MTBC (%) LAMP
**Z-N staining**		
+	+	38/38 (100.0 %)
+	–	6/10 (60.0 %)
–	+	19/22 (86.4 %)
–	–	7/60 (11.7 %)
**Fluorescence microscopy**		
+	+	41/41 (100 %)
+	–	8/12 (66.7 %)
–	+	16/19 (84.2 %)
–	–	5/58 (8.6 %)
+	+	41/41 (100 %)

**+** means samples tested positive.

- means samples tested negative.

### *3.4* Comparison of results of TB-LAMP assay with fluorescence microscopy and culture results

In both Fluorescence microscopy positive and Culture positive 41 cases, all cases were positive by LAMP assay (100 %). Nineteen (19) cases were Fluorescence microscopy negative but Culture positive. Out of these 19 cases, 16 (84.2 %) cases were positive by LAMP assay. In both Fluorescence microscopy and Culture negative 58 cases, 5 (8.6 %) cases were LAMP assay positive (Table-2).

### *3.5* Diagnostic performance of TB-LAMP assay in comparison with Z-N staining, fluorescence microscopy and qPCR considering culture as a gold standard

Performance characteristics of AFB smear by Z-N staining, fluorescence microscopy by Auramine-O staining, qPCR for MTBC and LAMP assay were done considering culture as a gold standard. It was seen that out of 60 culture positive cases, 38(63.3 %) were true positive and 22(36.7 %) were false negative by AFB smear, 41(68.3 %) cases were true positive and 19(31.7 %) were false negative by fluorescence microscopy, 57(95 %) cases were true positive and 3(5 %) cases were false negative by LAMP assay, 58(96.7 %) cases were true positive and 2(3.3 %) were false negative by qPCR for MTBC.

Out of 70 culture negative cases, 10 (14.3 %) cases were false positive and 60(85.72 %) cases were true negative by Z-N staining; 12(17.14 %) cases were false positive and 58(82.86 %) cases were true negative by Fluorescence microscopy, 13 (18.57 %) cases were false positive and 57(81.43 %) cases were true negative by LAMP assay, 16(22.85 %) cases were false positive and 54(77.14 %) cases were true negative by qPCR for MTBC. The sensitivity, specificity, positive predictive value and negative predictive value of AFB smear by Z-N staining were 63.3 % 85.7 %, 79.2 % and 73.2 % respectively. The sensitivity, specificity, positive predictive value and negative predictive value of Fluorescence microscopy were 68.3 %, 82.9 %, 77.4 % and 75.3 % respectively. The sensitivity, specificity, positive predictive value and negative predictive value of qPCR for MTBC were 96.7 %, 77.1 %, 78.4 % and 96.4 % respectively. The sensitivity, specificity, positive predictive value and negative predictive of LAMP assay were 95 %, 81.4 %, 81.4 % and 95 % respectively. The kappa coefficients of AFB smear by Z-N staining, fluorescence microscopy, qPCR for MTBC and LAMP assay were 0.50, 0.52, 0.76 and 0.73 respectively (Table-3).

**Table 3:** Diagnostic performance of TB-LAMP assay in comparison with Z-N staining, Fluorescence microscopy and R-T PCR considering culture as a gold standard.

Methods	Result	Culture result		S (95 % CI)	Sp (95 % CI)	PPV (95 % CI)	NPV (95 % CI)	kappa
		MTBC (+) (n = 60)	MTBC (−) (n = 70)					
Z-N staining	positive	38	10	63.3 %	85.7 %	79.2 %	73.2 %	0.50
		(63.3 %)	(14.3 %)					
	negative	22	60					
		(36.7 %)	(85.7 %)					
Fluorescence microscopy	positive	41	12	68.3 %	82.9 %	77.4 %	75.3 %	0.52
		(68.3 %)	(17.1 %)					
	negative	19	58					
		(31.7 %)	(82.9 %)					
LAMP	positive	57	13	95 %	81.4 %	81.4 %	95 %	0.76
		(95.0 %)	(18.6 %)					
	negative	3	57					
		(5.0 %)	(81.4 %)					
qPCR MTBC	positive	58	16	96.7 %	77.1 %	78.4 %	96.4 %	0.73
		(96.7 %)	(22.9 %)					
	negative	2	54					
		(3.0 %)	(77.1 %)					

S= Sensitivity.

Sp = Specificity.

PPV = positive predictive value.

NPV = negative predictive value.

Sensitivity, Specificity, PPV and NPV were calculated by chi-square test.

Agreement between TB-LAMP and qPCR has been measured by Cohens' kappa formula.

Kappa coefficients are shown in number according to interpretive criteria as follows: 0.21 to 0.4 (fair); 0.41 to 0.60 (moderate); 0.61 to 0.80 (substantial); 0.81 to 1.0 (excellent).

### *3.6* Comparison with the LAMP assay and qPCR by correlation

Test agreement between LAMP assay and qPCR was excellent (0.84). Among 74 MTBC-positive samples in qPCR, LAMP assays were positive in 67(90.6 %) cases. All 10 NTM-positive cases detected by qPCR were found to be negative by LAMP assay (Table-4).

**Table 4:** Comparison with the TB -LAMP and qPCR by correlation.

qPCR (n)	LAMP Positive n (%)	LAMP Negative n (%)	Sensitivity	Specificity	PPV	NPV	kappa
MTBC (74)	67 (90.5 %)	7 (9.5 %)	90.5 %	94.6 %	95.7 %	88.3 %	0.84
NTM (10)	0 (0.0 %)	10 (100 %)					
Negative (46)	3 (6.5 %)	43 (93.5 %)					
Total (130)	70 (53.9 %)	60 (46.2 %)					

PPV = positive predictive value.

NPV = negative predictive value.

Sensitivity, Specificity, PPV and NPV were calculated by chi square test.

Agreement between TB-LAMP and qPCR has been measured by Cohens' kappa formula.

Kappa coefficients are shown in number according to interpretive criteria as follows: 0.21 to 0.4 (fair); 0.41 to 0.60 (moderate); 0.61 to 0.80 (substantial); 0.81 to 1.0 (excellent).

## 4 Discussion

A major limitation while fighting against pulmonary TB is the unavailability of cheap, fast and accurate detection methods which can be used in resource-limited settings. Recently, TB-LAMP assay, a rapid and easy technique has been developed, expected to become the primary and noble molecular method to detecting MTBC [16]. The study aimed to evaluate the performance of multiplex loop-mediated isothermal amplification (LAMP) assay & compare it with MTB/NTM qPCR, culture, Z-N staining and fluorescence microscopy.

The overall performance of TB-LAMP assay was superior to both AFB staining and Auramine-O staining. The rate of positivity of TB-LAMP assay was more than Z-N staining, fluorescence microscopy and culture. TB-LAMP assay detected 22 (17.0 %) cases more than Z-N staining and 17 (13.1 %) cases more than fluorescence microscopy and 10 (7.7 %) cases more than culture. The identification rate of multiplex LAMP assay was close to that of qPCR. Our findings are comparable with the study performed in Thailand by Phetsuksiri et al., in 2020 and the study performed at three hospital laboratories in Luskain, Zambia [13,20].

In both smear positive and culture positive cases the rate of detection of MTBC by multiplex TB-LAMP assay was excellent. It was also recommendable in fluorescence microscopy negative and culture positive cases. LAMP assay was positive (100 %) in all fluorescence microscopy-positive and culture-positive cases. Among 19 fluorescence-microscopy negative but culture-positive cases, 16 (84.1 %) cases were positive by LAMP assay. Similar studies in India and Thailand showed comparable findings with this study [5,13]. TB-LAMP assay didn't miss any of the 2+ and 3+ samples. The LAMP assay rate of positivity was also increased with intensity of both Z-N staining grading and fluorescence microscopy grading. Which is in concordance with a previous study in South Korea [16].

The LAMP assay sensitivity was 95.0 % which was higher than both Z-N staining (63.3 %) and fluorescence microscopy (68.3 %). This study showed almost similar results with other studies which revealed superior performance of LAMP assay than smear microscopy [13,16]. The agreement between culture and Z-N staining as well as fluorescence microscopy were moderate whereas the agreemen between culture and LAMP as well as qPCR were good. A study in Thailand found similar results to our study [13].

Though the specificity of TB- LAMP (81.4 %) was less than specificity of Z-N staining (85.7 %) as well as fluorescence microscopy (82.9 %), similar findings were revealed in another study [6]. The specificity of the LAMP assay became lower here because this study considered culture as gold standard to see the performance of the LAMP assay as culture-positive cases were only 60 (46.2 %). This may be due to delayed sample delivery by the patient which may cause death of cells during inoculation in culture media [13]. The solid culture media may also be contaminated and the rate is 2 %–5 % [21]. These could fail culture growth. In our study, TB-LAMP had missed some culture-positive samples. This may be explained by the loosing of DNA during the extraction procedure as high quality, pure DNA extraction protocol is necessary for successful molecular detection methods [22]. As LAMP assay was performed in unprocessed samples there is a chance of getting low DNA volume during amplification. Nevertheless, negative NAAT results in culture-positive cases shouldn't be ignored and require further evaluation.

In this study 10 Z-N staining positive samples and 12 fluorescence microscopy positive samples failed to give growth in culture. It is not unusual that specimen decontamination before culture using 2 % NaOH could destroy weakly growing mycobacteria, thereby lowering culture sensitivity [18]. A study in Bangladesh revealed 4 % false positive cases by Z-N staining and 8 % false positive cases by fluorescence microscopy [23]. Another study in Thailand found 28 smear-positive but culture-negative cases out of 252 samples [13]. Results of above mentioned studies are consistent with our study.

Correlation between results of qPCR and LAMP assay has shown sensitivity, specificity, PPV and NPV of LAMP assay based on qPCR were 90.5 %, 94.6 %, 95.7 % and 88.3 % respectively. Ten NTM positive samples by qPCR were negative by LAMP assay. There was excellent agreement between LAMP assay and qPCR (k = 0.84). A similar study performed in South Korea found LAMP assay sensitivity and specificity of 85 % and 100 % respectively compared with qPCR. In that study, the agreement between these two tests was also excellent, which are inconsistent with this study [16].

To assess specificity of TB-LAMP here we used samples from clinically suspected cases that potentiate the study. Small sample size is limitation. Due to time and budget constraints, a population-based study could not be conducted using a generalized strategy. Despite promising preliminary results, further large-scale validation studies involving diverse patient populations, including those with extrapulmonary tuberculosis and HIV coinfection, are necessary to evaluate the assay's performance characteristics, clinical utility, and impact on patient management outcomes. These limitations underscore the importance of continued research, validation and refinement of multiplex LAMP assays for tuberculosis diagnosis, with the ultimate goal of improving patient care and disease control efforts.

The high positive rate of TB-LAMP assay and isolation of MTBC from both AFB staining negative and Auramine-O staining negative samples prove the superiority of TB-LAMP assay over either microscopy technique. According to recommendation of World Health Organization (2016), with the evidence of this study, we speculate that multiplex TB-LAMP assay being a molecular method, can replace microscopy at resource-poor settings/hard-to-reach areas and will help NTP in Bangladesh to increase detection rate of MTBC. TB- LAMP can be a useful screening method of tuberculosis for high-risk groups (prisoners, immigrants, etc.). As the country is still now using microscopy for initial detection method. Synergistic and complementary use of TB-LAMP assay with other molecular methods would help to replace the microscopy. Physicians can rely on TB-LAMP positivity of presumptive PTB cases as it is a rapid molecular method and can start treatment as soon as the result is available. The major outcome of this study highlighted that TB-LAMP assay could be a powerful tool for earlier diagnosis of pulmonary tuberculosis which can pave the way to initiating treatment for the clinicians as well as interruption of highly infectious disease like tuberculosis.

## 5 Conclusions

Multiplex TB-LAMP assay has shown high sensitivity (95 %) compared with both Fluorescent microscopy (68.3 %) and Z-N staining (63.3 %). The overall performance of multiplex TB-LAMP assay is almost similar to that of qPCR. This indicates its ability to detect more TB cases like any other molecular technique if it is endorsed as diagnostic technique for presumptive PTB cases. A substantial rate (86.4 %) of TB-LAMP positive but AFB negative cases and the proportion (84.2 %) of Fluorescent microscopy negative but TB-LAMP positive cases that gave positive growth of *Mycobacterium tuberculosis* complex upon culture are significant. So, the findings of this study support an urgent need to endorse TB-LAMP assay for screening of clinically suspected cases of pulmonary tuberculosis in resource poor settings/hard to reach areas of low muddle income country like Bangladesh.

## CRediT authorship contribution statement

**Md Zaber:** Writing – original draft, Validation, Software, Methodology, Investigation, Data curation. **Fahmida Hoque:** Writing – original draft, Software, Investigation, Data curation. **Ishraque Monir Paean:** Writing – original draft, Software, Data curation. **Shirin Tarafder:** Writing – review & editing, Validation, Supervision, Methodology, Investigation, Conceptualization.

## Author agreement

The submitted manuscript has been approved by the authors. It is original work done by the authors, was not published before and isn't submitted elsewhere for publication.

## Ethical approval

Approval of the research protocol was taken from Institutional Review Board (IRB) of BSMMU (No. BSMMU/2022/6769) on 07/07/2022. Written informed consent was obtained from all the participants.

## Data availability statement

Data will be made available on request from the corresponding author.

## Author agreement

The submitted manuscript has been approved by the authors. It is original work done by the authors, was not published before and isn't submitted elsewhere for publication.

## Declaration of competing interest

The authors declare that they have no competing financial interests or personal relationships that could have appeared to influence the work reported in this paper.
