# Supplementary material for: Evaluation of Multiplex loop-mediated isothermal amplification assay for the detection of Mycobacterium tuberculosis complex from clinically suspected cases of pulmonary tuberculosis
Source: Heliyon. 2024 Oct 24;10(21):e39847. doi: 10.1016/j.heliyon.2024.e39847 (PMC11550662; doi:10.1016/j.heliyon.2024.e39847)
Supplement: Multimedia component 1 [file mmc1.docx]

**Appendices (Supplementary Material):**

**The following are the supplementary data related to this article**

**APPENDIX- I**

**Photographs**

**
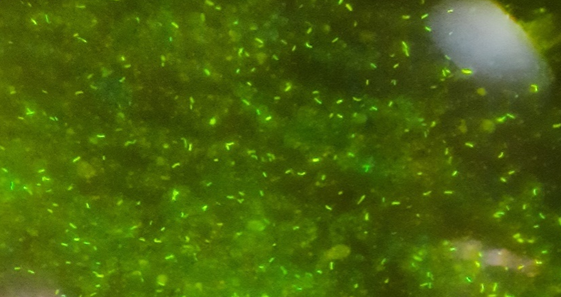
**

**Supplementary Figure 1 : Acid fast bacillus in Fluorescence microscopy with Auramine-O stain showing green fluorescent bacilli.**

**
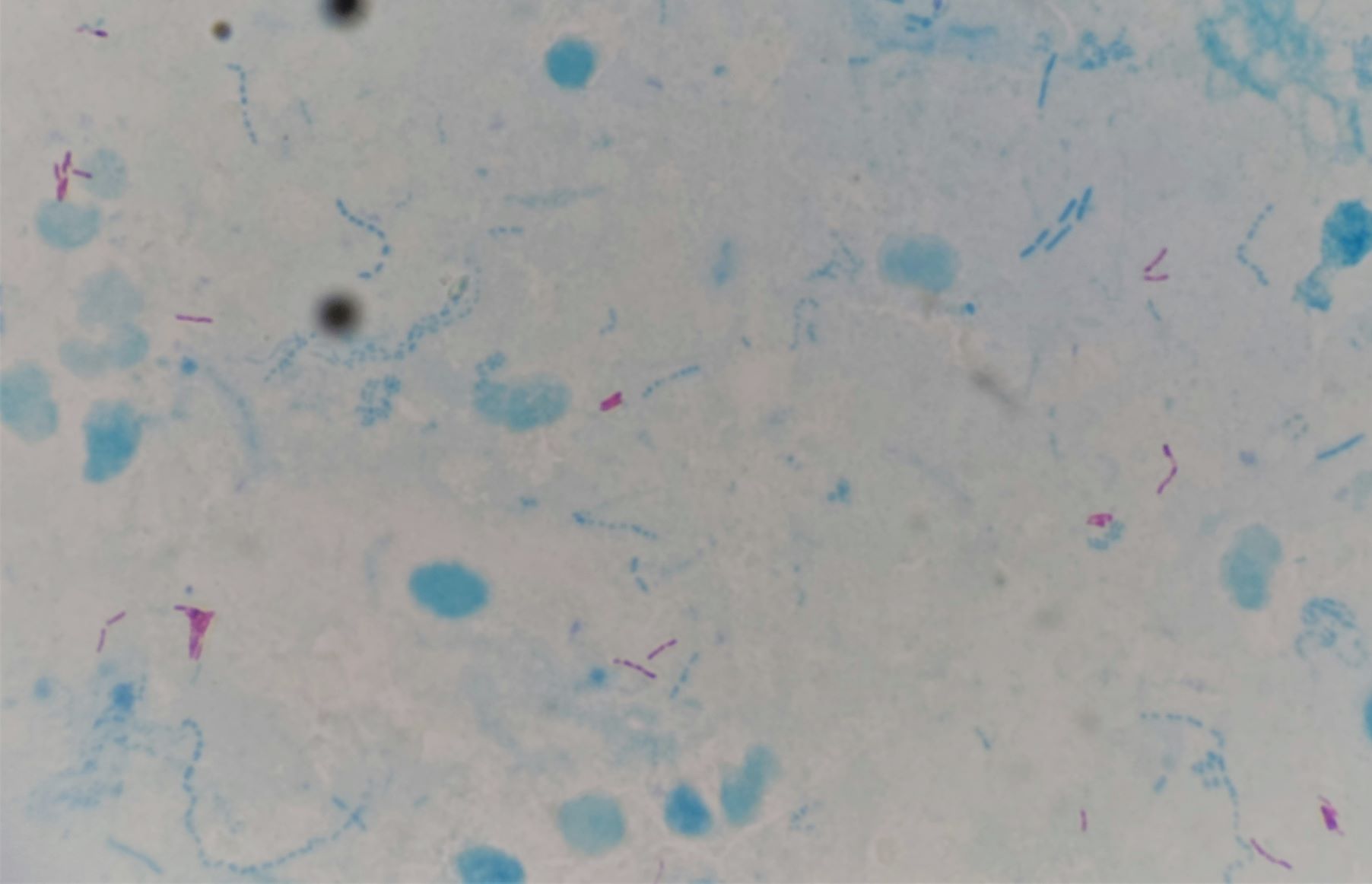
**

**Supplementary Figure 2: Acid fast bacilli in Z-N staining smear prepared from clinically suspected sputum sample.**

**
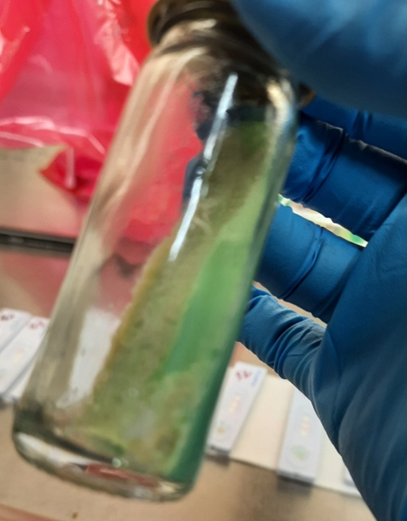
**

**Supplementary Figure 3: Growth of Mycobacterium tuberculosis colony in L-J media**

**
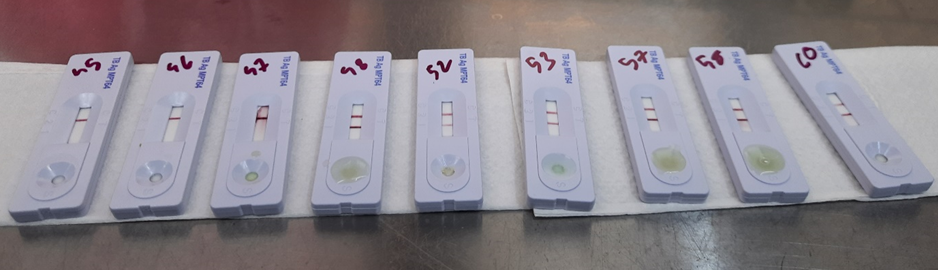
**

**Supplementary Figure 4: MPT 64 test to showing positive test line (T) and positive control line (C)**


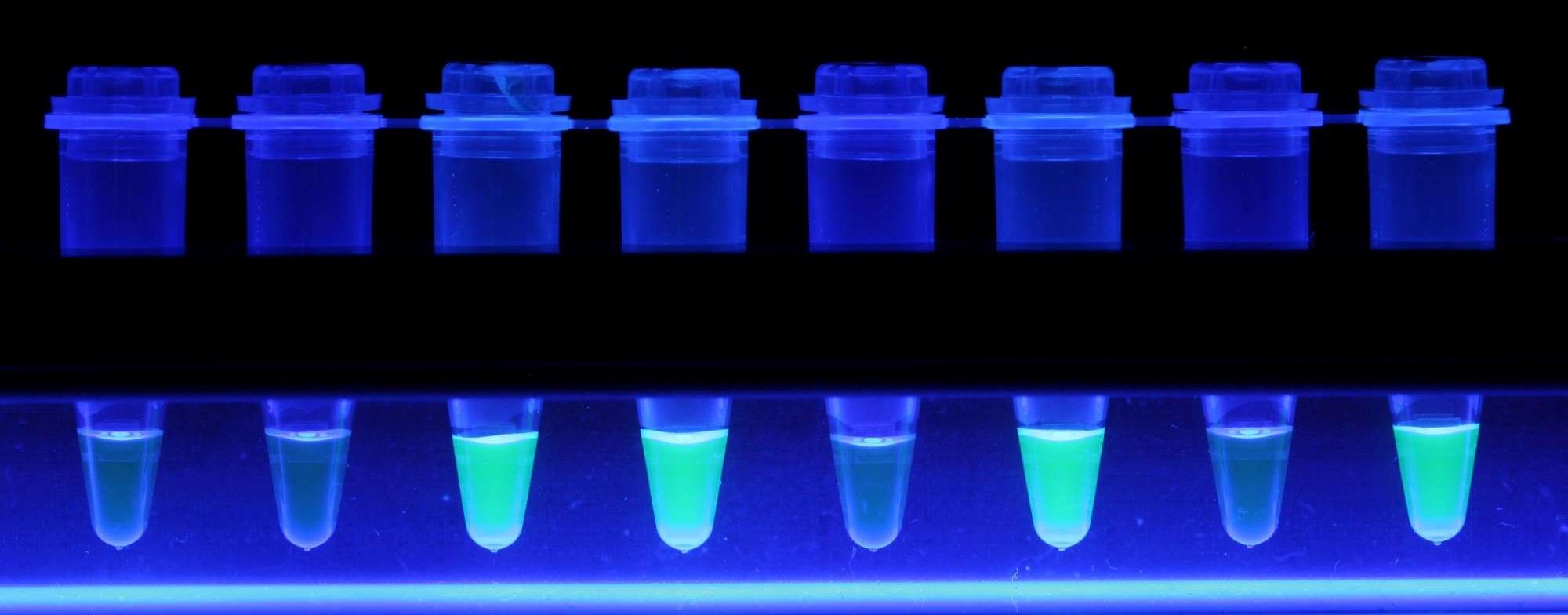


**Supplementary Figure 5: Multiplex LAMP assay for MTBC with HumaLoop T showing positive MTBC amplification by green fluorescence.**

**
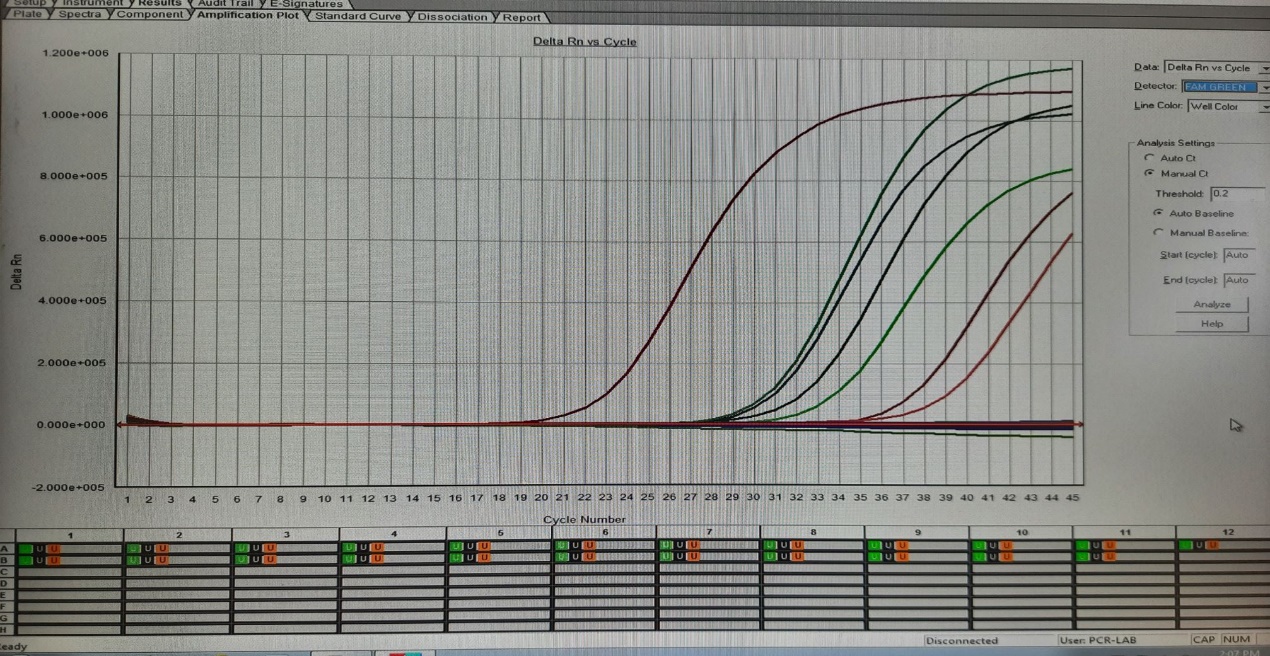
**

**Supplementary Figure 6 : Multiplex RT-PCR amplification plot analysis curve of MTBC detection**

**
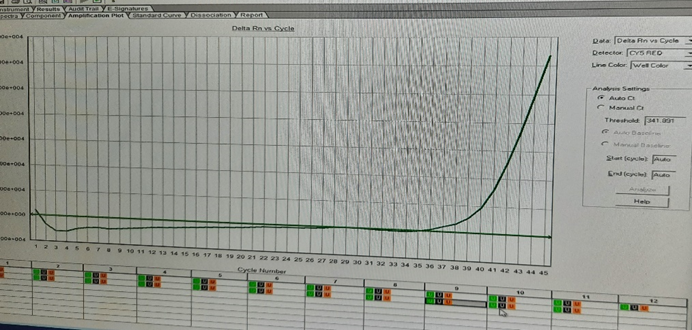
**

**Supplementary Figure 7: Multiplex RT-PCR amplification plot analysis curve of NTM detection**

**APPENDIX- II**

**Data of all samples in Excel data sheet**
